# Charge Carrier Transport Behavior and Dielectric Properties of BaF_2_:Tb^3+^ Nanocrystals

**DOI:** 10.3390/nano10010155

**Published:** 2020-01-16

**Authors:** Xiaoyan Cui, Tingjing Hu, Huangyu Wu, Junkai Zhang, Lihua Yang, Xin Zhong, Xiaoxin Wu, Jingshu Wang, Xuefei Li, Jinghai Yang, Chunxiao Gao

**Affiliations:** 1 Key Laboratory of Functional Materials Physics and Chemistry of the Ministry of Education, National Demonstration Center for Experimental Physics Education, Jilin Normal University, Siping 136000, China; xycuimail@163.com (X.C.); huangyuwu@126.com (H.W.); junkaizhang126@126.com (J.Z.); ylh@calypso.cn (L.Y.); zhongxin@calypso.cn (X.Z.); xiaoxin.wu@hotmail.com (X.W.); jingshuwang126@126.com (J.W.); xuefeili163@163.com (X.L.); jhyang1@jlnu.edu.cn (J.Y.); 2 State Key Laboratory of Superhard Materials, Jilin University, Changchun 130012, China; chunxiaogao126@126.com

**Keywords:** nanocrystals, electrical properties, dielectric behavior, ion conduction

## Abstract

The charge carrier behavior and dielectric properties of BaF_2_:Tb^3+^ nanocrystals have been studied by alternating current (AC) impedance spectroscopy. The electron and ion coexist in the transport process. The F^−^ ion’s contribution to the total conduction increases with the doping concentration up to 4% and then decreases. Tb doping leads to the increase of defect quantities and a variation of charge carrier transport paths, which causes the increase of the ion diffusion coefficient and the decreases of bulk and grain boundary resistance. When the Tb-doped concentration is higher than 4%, the effect of deformation potential scattering variation on the transport property is dominant, which results in the decrease of the ion diffusion coefficient and increases of bulk and grain boundary resistance. The conduction properties of our BaF_2_:Tb^3+^ nanocrystals are compared with previous results that were found for the single crystals of rare earth-doped BaF_2_. Tb doping causes increases of both the quantity and the probability of carrier hopping, and it finally leads to increases of BaF_2_ nanocrystals’ permittivity in the low frequency region.

## 1. Introduction

BaF_2_ is an important material that is widely applied as a host for optically active centers [1,2,3,4,5] and as an electrolyte for solid-state electrochemical devices [6,7]. Meanwhile, BaF_2_ is an ideal high-density luminescent material that has been widely used in gamma ray and elementary particle detectors [8,9]. Though the doping of appropriate impurity elements into the lattice, BaF_2_ has a better ionic conductivity and becomes an important candidate material for high temperature batteries, fuel cells, chemical filters, and sensors [10]. Therefore, BaF_2_ electrical and dielectric properties are attractive research subjects. Now, there are mainly two methods that are used to modulate the transport performance of alkaline earth fluorides: One uses a nanoscale sample because nanostructure can change the conduction paths of a charge carrier [11]; the other is the doping of a rare earth ion that can be easily incorporated as a dopant in a crystal lattice and results in the variation of point defect quantity [12,13,14].

Several studies have been conducted on the conductivity of rare earth-doped BaF_2_ [12,13,14]. Ivanov-Shits et al. [12] prepared Ba_1−*x*_R*_x_*F_2+*x*_ single crystals with different rare earth elements (R) and with various compositions. For all rare-earth elements, the conductivity increased with *x*; to some specific rare earth elements, it was saturated at high concentration. Similar results were reported by Sorokin et al. [13] and Wapenaar et al. [14]. However, all of them carried out their investigations on single crystals [12,13,14]. Few works have been made on the nanostructure sample. Therefore, it is necessary to conduct a detailed investigation on the transport properties of nanoscale, rare earth-doped BaF_2_, such as the carrier type, the individual contribution of grain and the grain boundary to transport behavior, and the dielectric property. In this investigation, Tb-doped BaF_2_ nanocrystals were investigated by X-ray diffraction (XRD), energy dispersive spectrometry (EDS), transmission electron microscopy (TEM), and alternating current (AC) impedance spectroscopy. The carrier transport behavior and dielectric properties are discussed.

## 2. Materials and Methods 

The sample synthesis process has been described in our previous reports [15,16,17,18,19,20]. The samples were synthesized as the following: 16.8 mL of oleic acid, 48 mL of ethanol and 0.4 g of sodium hydroxide were mixed and stirred for 10 min; then, 0.008 mol of barium nitrate was dissolved into 20 mL water, which was add into the above solution and stirred for 10 min; finally, 0.012 mol of sodium fluoride that was dissolved into 20 mL of water was added and stirred for 60 min. The above mixed solution was put into a 100 mL autoclave and kept at 180 °C for 24 h; the reactants were collected by centrifugation. The Tb doping concentrations were 2, 4, 6, 8, 10 mol%. The samples were characterized by XRD, EDS and TEM. The AC impedance was conducted by parallel plate electrodes. The sample diameter was 6 mm, and its thickness was 1 mm. The AC impedance was measured by a Solartron 1260 (Hampshire, UK) connected with Solartron 1296 (Hampshire, UK). The detail measurement parameters have illustrated in previous works [21]. The input voltage was 0.1 V and the frequency region was 0.1–10^7^ Hz.

## 3. Results and Discussion

**Figure 1:**
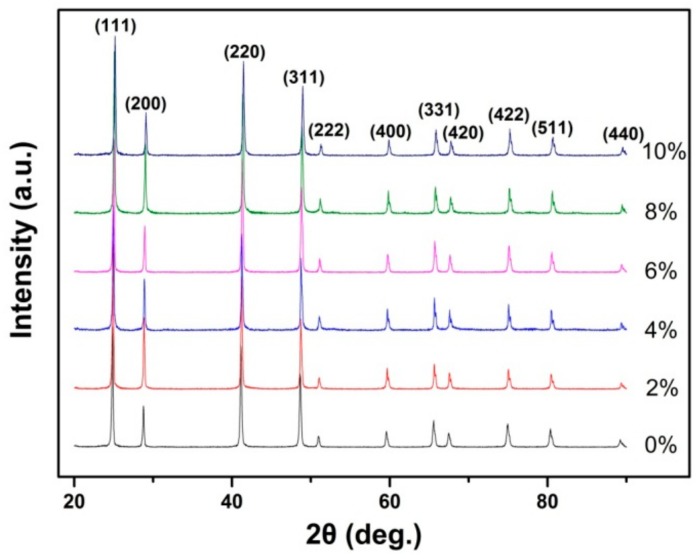
The XRD patterns of the BaF_2_:Tb^3+^ nanocrystals.

**Figure 2:**
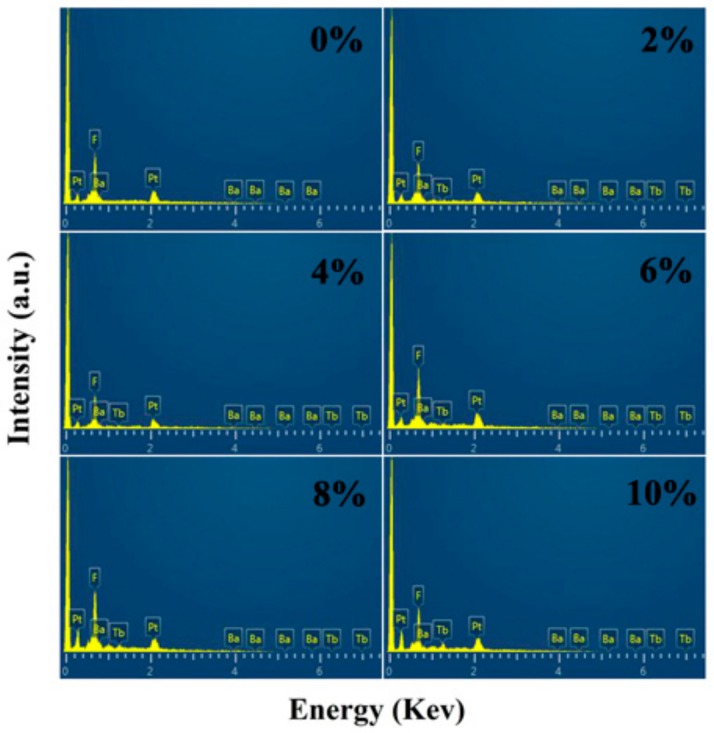
The energy dispersive spectrometry (EDS) the spectra of BaF_2_:Tb^3+^ nanocrystals.

**Figure 3:**
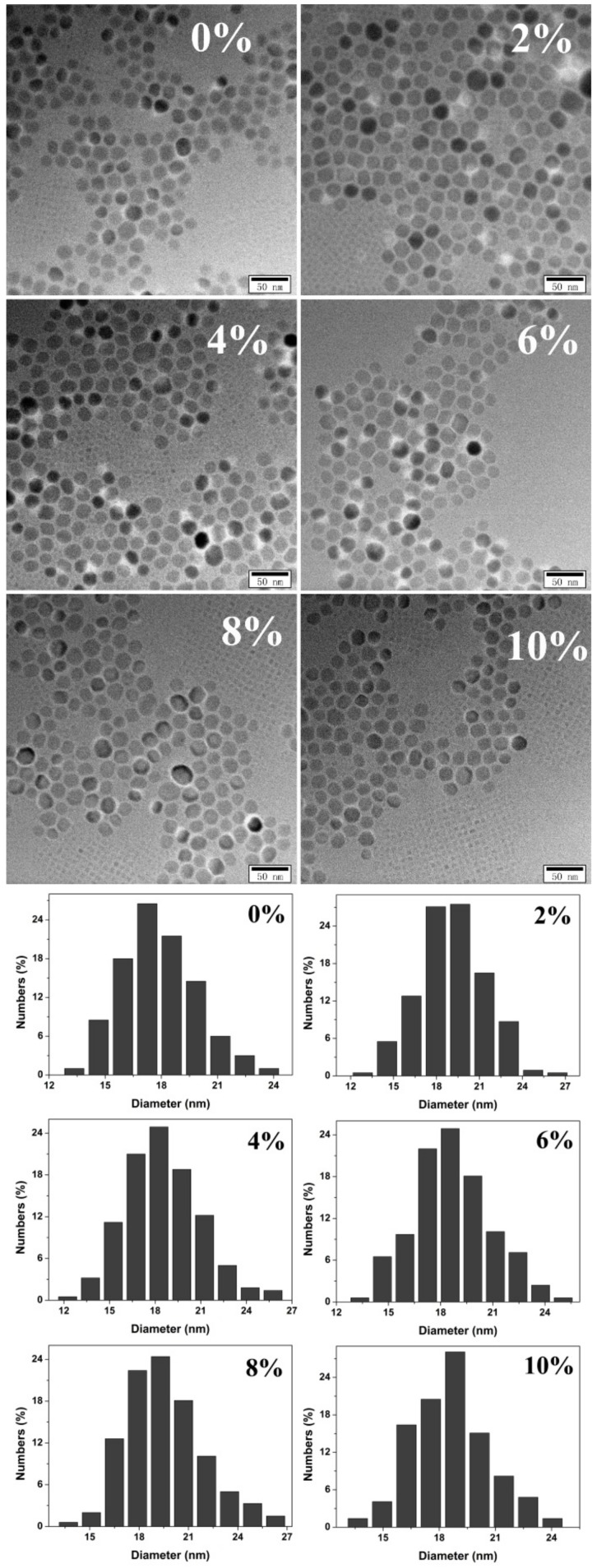
The TEM image and size distribution histogram of the BaF_2_:Tb^3+^ nanocrystals.

All the XRD patterns (Figure 1) could be indexed as a cubic BaF_2_ phase (JCPDS Card No. 04-0452), which indicates that the structure of the BaF_2_ nanocrystals was unchanged after doping. From the XRD results, it could be seen that the XRD peaks shifted towards a higher angle. The Tb^3+^ ionic radius (0.092 nm) was smaller than the Ba^2+^ ionic radius (0.135 nm); therefore, Tb doping led to the shifting of the XRD peaks towards a higher angle. The lattice parameters that were yielded by refining the XRD pattern are listed in Table 1. The emergence of the Tb peaks in the Tb doping EDS spectra (Figure 2) confirm that the Tb^3+^ was successfully doped into the BaF_2_ crystal lattice. The TEM results (Figure 3) exhibit that the shape of the particle of all the samples was cubic, and its size was about 19 ± 4 nm. By analyzing the size distribution histogram, it was found that the average size of the Tb-doped BaF_2_ was larger than the average size of the un-doped sample, which indicates that the Tb doping promoted the sample’s crystal growth.

**Figure 4:**
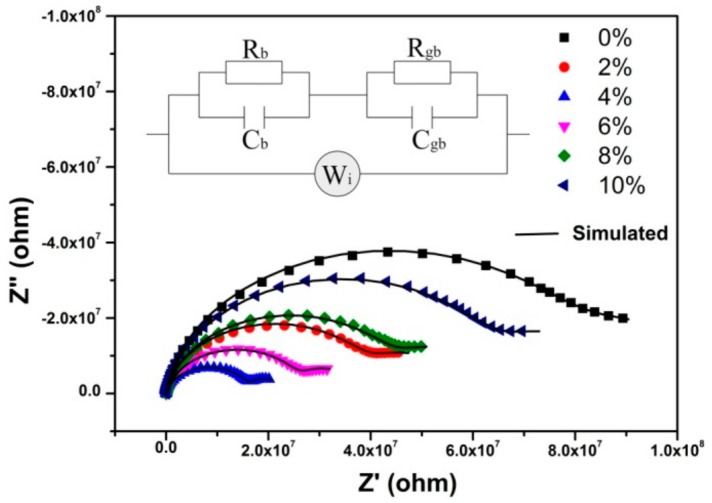
The complex impedance curves of the BaF_2_:Tb^3+^ nanocrystals. The inset is the equivalent circuit, *R_g_* and *C_g_* are the bulk resistance and capacitance, *R_gb_* and *C_gb_* are the grain boundary resistance and capacitance, and *W_i_* is the Warburg element.

**Figure 5:**
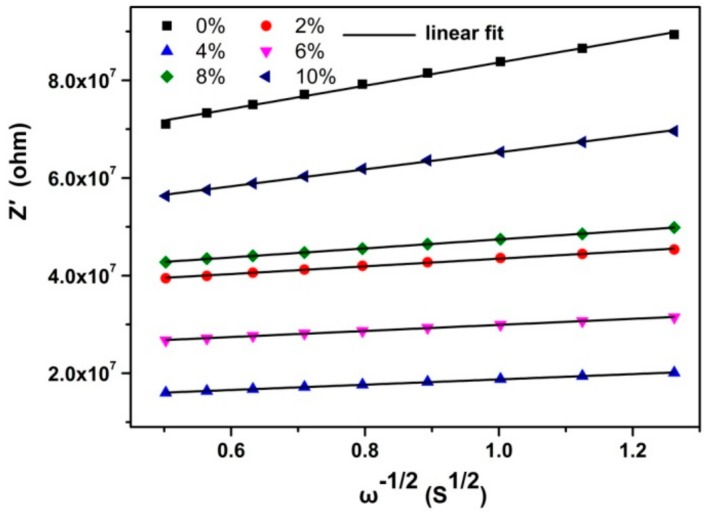
*Z′* vs. *ω*^−1/2^ in the low frequency region of the BaF_2_:Tb^3+^ nanocrystals.

**Figure 6:**
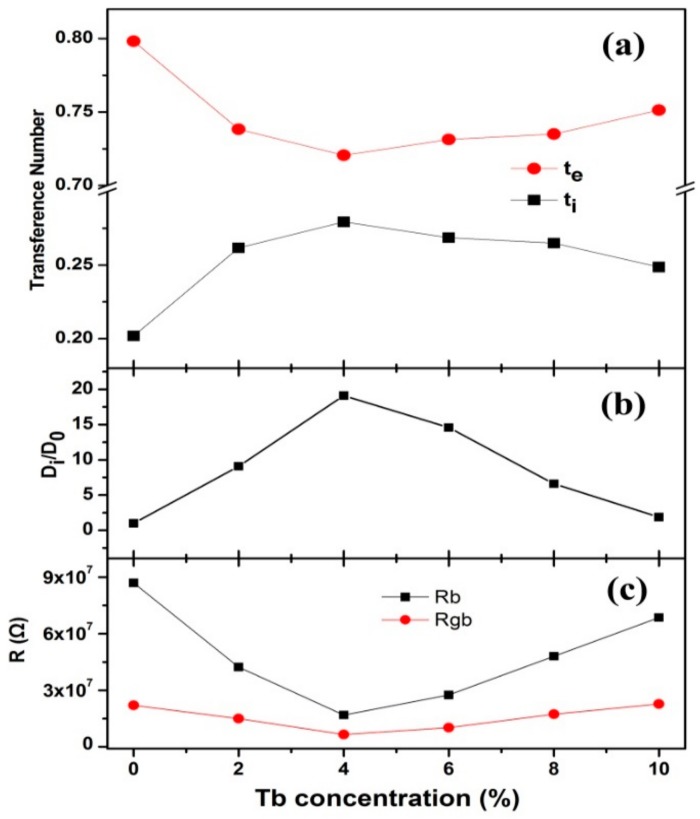
The transference number of the ion and the electron (**a**), the ion diffusion coefficient (**b**), and the bulk and grain boundary resistance (**c**) of the BaF_2_:Tb^3+^ nanocrystals.

The complex impedance curves of the BaF_2_:Tb^3+^ nanocrystals are shown in Figure 4. To study their ionic transport property, the curves were replotted into *Z*′ vs. *ω^−1/2^* representation in the low frequency region, as shown in Figure 5. It can be seen *Z′* exhibited a linear relation with *ω^−^*^1/2^ in all the samples, indicating the existence of F^−^ ion diffusion through the grain boundaries at low frequencies. The impedance spectra were simulated by the equivalent circuit as the inset of Figure 4. According to the analytical methods in our previous report [22], the ion transference number (*t_i_*) and electron transference number (*t_e_*) can be obtained by the following formulas:

$$t_{i}\;=\;\left(R_{2}\;-\;R_{1}\right)/R_{2}$$

$$t_{e}\;=\;R_{1}/R_{2}$$

where *R*_1_ and *R*_2_ are the *X*-axis intercepts of the complex impedance curves. The ion diffusion coefficient can be obtained by

$$D_{i}\;=\;0.5{\left(\frac{RT}{AF^{2}\sigma C}\right)}^{2}$$

where the Warburg coefficient (*σ*) was obtained by linear fitting *Z′*~*ω^−1/2^* plot. The ion and electron transference number, the ion diffusion coefficient, the bulk and grain boundary resistance are shown in Figure 6.

It can be seen that the electron transference number was larger in all samples (Figure 6a), while the ion transference number increased with the Tb doping concentration up to 4% and then decreased. From Figure 6b,c, it can be seen that the ion diffusion coefficient increased with the Tb-doped concentration up to 4% and then decreased. The bulk and grain boundary resistance decreased with the Tb-doped concentration and reached minimum values at 4%. The variations of transport parameters can be explained as following: Tb doping led to the defect quantity increasing and the charge carrier transport paths varying, which resulted in the charge carrier concentration increasing and the activation energy decreasing; these finally caused the increase of the ion diffusion coefficient and the decrease of the bulk and grain boundary resistance. When the Tb-doped concentration was higher than 4%, the defects aggregated into clusters, which resulted in the carrier concentration remaining almost constant. Therefore, the defect variation effect on the carrier transport was small. As Tb doping increased, the deformation potential scattering was enhanced, which made the charge carrier transport more difficult. The effect of deformation potential scattering enhancement was dominant. All of these finally resulted in the decrease of the ion diffusion coefficient and the increase of bulk and grain boundary resistance.

According to previous reports, the ion is the only charge carrier in the single crystals of rare earth-doped BaF_2_ [12,13,14], though the charge carriers of our BaF_2_:Tb^3+^ nanocrystals included both an ion and an electron. For both single crystals and nanocrystals, rare earth-doped concentration has a critical point. Before the critical point, the conduction of both the single crystals and nanocrystals increased with the doping concentration. When the doping concentration was larger than the critical value, the conduction variations were different: for the single crystals, the conduction increased slowly or kept almost constant [12,13,14], while for our nanocrystals, the conduction decreased with the increased doping concentration. We thought these differences originated from the composition differences between the single crystals and nanocrystals; with the crystal size decreasing, the proportion of the surfaces (interfaces) increased and the grain boundary properties varied, which made the charge carrier types and the doping effect on the transport behavior of the nanocrystals different from those of the single crystals.

**Figure 7:**
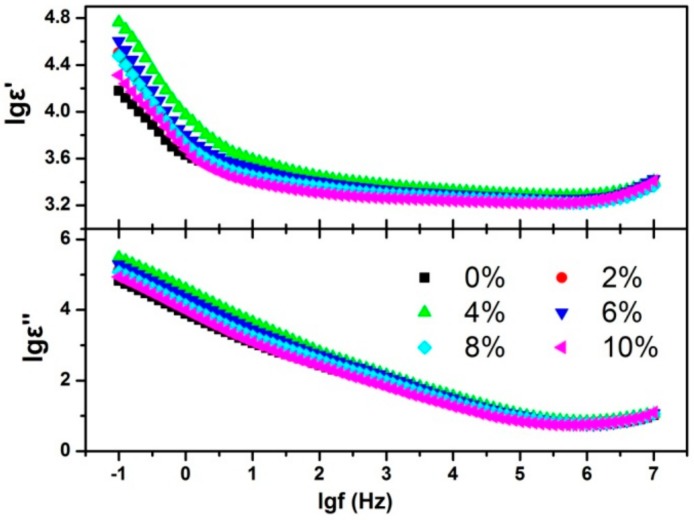
The frequency dependence of the real part (*ε′*) and imaginary part (*ε″*) of the permittivity.

The complex permittivity was also investigated, and the data of this investigation are shown in Figure 7. At a high frequency (*f* > 1 MHz), the *ε′* increased with the increasing frequency and the dielectric loss peak began to form, which was caused by the dipole orientation polarization. In the low frequency region, *ε′* and *ε″* increased sharply with the decreasing frequency. This dispersion phenomenon indicates the carriers (electrons and interstitial fluoride ions) were hopping under the electric field [13,14,23]. With the frequency increasing, the carrier hopping frequency was smaller than the applied electric field frequency, which led to the *ε′* and *ε″* decreasing. The *ε′* and *ε″* values of the Tb doping BaF_2_ nanocrystals in the low frequency was higher than that of the un-doped sample. This phenomenon can be explained as being due to the different valences between Tb^3+^ and Ba^2+^. Tb doping results in the formation of interstitial fluoride ions [13,14], which causes the ion quantity that participates in the hopping process to increase. Meanwhile, Tb doping decreases the energy barrier of carrier hopping [13,14], which means that the carrier hopping probability increased. Therefore, Tb doping caused both the quantity and the probability of carrier hopping to increase, which finally led to the BaF_2_ nanocrystals’ permittivity increasing in the low frequency region.

## 4. Conclusions

The charge carrier behavior and dielectric properties of Tb-doped BaF_2_ nanocrystals were studied with AC impedance spectroscopy. The electron and the ion coexisted in the transport process. The F^−^ ion’s contribution to the total conduction increased with the doping concentration up to 4% and then decreased. Tb doping led to the increase of defect quantity and the variation of charge carrier transport paths, which caused an increase of the ion diffusion coefficient and decreases of bulk and grain boundary resistance. When the Tb-doped concentration was higher than 4%, the effect of deformation potential scattering variation on the transport property was dominant, which resulted in a decrease of the ion diffusion coefficient and increases of the bulk and grain boundary resistance. Following a comparison with the single crystals results, it can be concluded that decreasing crystal size caused variations of surface (interfaces) proportion and grain boundary properties, which made the charge carrier types and the doping effect on the carrier transport behavior of the nanocrystals different from those of the single crystals. Tb doping caused increases of both the quantity and the probability of carrier hopping, finally leading to increases of BaF_2_ nanocrystal permittivity in the low frequency region. 

## Figures and Tables

**Table 1:** The lattice parameters that were yielded by refining the XRD pattern.

Tb-Doped Concentration	Lattice Constant (Å)	Cell Volume (Å^3^)
0%	6.2025	238.619
2%	6.1945	237.696
4%	6.1883	236.991
6%	6.1818	236.238
8%	6.1700	234.887
10%	6.1545	233.127
